# Safety and efficacy of polymyxin B in multidrug resistant Gram-negative severe sepsis and septic shock

**DOI:** 10.4103/0972-5229.45074

**Published:** 2008

**Authors:** Suresh Ramasubban, Ayanava Majumdar, Purnendu Sekhar Das

**Affiliations:** **From:** Department of Critical Care Medicine, Apollo Gleneagles Hospital, Kolkata, W. Bengal, India

**Keywords:** Acute renal failure, Gram-negative organisms, multidrug resistant, polymyxin B, severe sepsis and septic shock

## Abstract

**Background and Aims:**

The emergence of multidrug resistant strains of Gram-negative bacteria, especially the lactose nonfermenters like *Pseudomonas* and *Acinetobacter*, in the intensive care units have prompted renewed worldwide interest in the polymyxins. However, perceived nephrotoxicity has been a major vexation limiting their early and regular use in severe sepsis. This study was conducted to assess the safety and efficacy of polymyxin B in patients with severe sepsis and septic shock.

**Materials and Methods:**

Forty-five patients with sepsis admitted in our medical-surgical intensive care units were identified from pharmacy records to have received polymyxin B. We retrospectively reviewed the clinical and microbiologic outcomes as well as occurrence of renal failure temporally related to the use of intravenous polymyxin B.

**Results:**

polymyxin B was used in severe sepsis and septic shock with the isolated organism being resistant to other available antimicrobials or clinical deterioration despite carbapenem use. Overall mortality was 52% and among patients who received at least eight days of intravenous polymyxin B, 67% patients with initial septic shock and 62% with severe sepsis survived. The target multidrug resistant organism was cleared in 88% of subjects evaluated by repeat microbiologic testing. Acute renal failure developed in only two patients (4%).

**Conclusions:**

Polymyxin B has acceptable effectiveness against nosocomial multidrug resistant Gram-negative sepsis. The associated nephrotoxicity has been found to be significantly lower than previously reported even in patients with background renal impairment and established risk factors of renal failure.

## Introduction

Multidrug resistant (MDR) Gram-negative bacterial sepsis poses an increasingly daunting challenge in the critical care setting worldwide.[1–2] Especially the lactose nonfermenters *Pseudomonas* and *Acinetobacter*, which are opportunistic niche pathogens affecting primarily the critically ill and the immunocompromised, ubiquitous in the hospital environment and notorious for developing multiresistance to available antibiotics, are making management of sepsis not only difficult but also very expensive even for the industrialized countries.[3–5] Hence, the worldwide interest in the polymyxins, which have shown consistent efficacy against aerobic Gram-negative bacilli including *Pseudomonas* and *Acinetobacter* by their detergent-like cell-membrane-disrupting and antiendotoxin actions,[6–7] yet their use has been restricted as the last resort in cases of clinical or microbiologic resistance to other available antibiotics, owing to the reports of nephrotoxicity and neurotoxicity.[8–9]

We reviewed our recent experience in the use of intravenous polymyxin B against MDR Gram-negative organisms with particular attention to its nephrotoxicity in the critical care unit in the 350-bed tertiary-level multispecialty Apollo-Gleneagles Hospital situated in Kolkata.

## Materials and Methods

Forty-five patients were identified from pharmacy records who had received more than two doses of intravenous polymyxin B during the period March 2006 and June 2007. The medical records were reviewed retrospectively for demographic details, underlying diseases, presence, severity and source of sepsis, organisms isolated and their antibiotic sensitivity, length of stay in the ICU, dose, frequency and duration of polymyxin B, other antibiotics used, mechanical ventilation and creatinine levels, development of any new rash or neurological problems, and clinical outcomes.

The main outcomes of interest included resolution of sepsis, as well as microbiological response to polymyxin B, survival and renal failure temporally linked to the use of polymyxin B.

Sepsis was identified by the presence of systemic inflammatory response parameters like temperature, tachycardia, tachypnoea, leucocytosis, and/or leucopenia. Severe sepsis was defined as the presence of organ dysfunction and septic shock as hypotension unresponsive to fluids.[10] Lower respiratory tract secretions were considered pathological if these systemic signs were associated with radiological changes in chest X-ray. Renal failure was defined as acute increase in the serum creatinine level by >0.5 mg/dL above the baseline over 24 hours. Baseline serum creatinine was defined as the most recent value available at the start of the treatment with polymyxin B.

## Results

As detailed in Table 1, the study population comprised 45 critically ill patients with the mean age of the patients being 53 years and 56% being males. Mean duration of stay in the ICU was 38 days (range 6–92 days).

**Table 1:** Demography, clinical, and microbiologic details of 45 patients receiving polymyxin B

Details	Values*
Age (years)	
Mean	53
Range	19–82
Males	25 (56)
Sites of isolation of MDR organisms	
LRT	22 (49)
Surgical wound	10 (22)
Abdomen	7 (16)
Blood	5 (11)
Pleural fluid	4 (9)
Urine	3 (7)
Burn wound	2 (5)
Organisms	
*Pseudomonas*	20 (44)
*Acinetobacter*	19 (42)
*Pseudomonas + Acinetobacter*	2 (4)
None identified	4 (8)
Mechanical Ventilation	38 (84)
Septic shock at the start of Polymyxin B	19 (42)
Comorbidities	
Malignancy	13 (29)
Diabetes	6 (13)
Cardiac	5 (11)
CRF	4 (9)
COPD	4 (9)
ICH	3 (7)
Burns	2 (4)
Recent CABG	2 (4)
Pancreatitis	2 (4)
AIDP	2 (4)
Pemphigus vulgaris, Polytrauma,	1each (2)
Bomb blast inj, Corrosive esophagitis	

* Values expressed as numbers (percentages) of Patients; LRT, lower respiratory tract; MDR, multidrug resistant; ICH, intracranial hemorrhage; CRF, chronic renal failure; CABG, coronary artery bypass grafting; AIDP, acute inflammatory demyelinating polyneuropathy

Malignancy was the most prevalent underlying comorbidity (29%) followed by diabetes mellitus (13%) and chronic renal failure (9%).

Polymyxin B was used in severe sepsis with microbiological or clinical refractoriness to carbapenem antibiotics. Forty-two percent of the subjects were in septic shock and 84% of patients were receiving mechanical ventilation at the time of initiation of polymyxin B. Dose of 15,000 to 25,000 units/kg of ideal body weight in two divided doses per day was used. Total daily dose was reduced by 25% and 50% when the calculated creatinine clearance was 20–50 mL/min and <20 mL/min, respectively. The mean total daily dose used was 1.2 × 10^6^ units and the mean duration 7.6 days. The mean number of days between ICU admission and start of polymyxin was 22 days.

All patients received carbapenems, 90% received piperacillin–tazobactam or cefoperazone–sulbactam combination before polymyxin was used. All the patients received other potentially nephrotoxic agents like NSAIDs and diuretics. Fifty-two percent received Amikacin and 16% received Vancomycin [Table 2].

**Table 2:** Antibiotics use

Antibiotics	Values
Total daily dose of polymyxin B (10^6^ Units)	
Mean	1.2
Range	1–1.5
Duration of Polymyxin B use (days)	
Mean	7.6
Range	2–32
Additional Antibiotics	
Cephalosporins	42 (93)
Amoxicillin-Sulbactam	40 (89)
Carbapenems	35 (78)
Piperacillin-Tazobactam	30 (67)
Aminoglycosides	28 (62)
Metronidazole	24 (53)
Fluoroquinolones	20 (44)
Aztreonam	20 (44)
Macrolides	14 (31)
Fluconazole	13 (29)
Vancomycin	7 (16)
Linezolid, Tetracyclines, Chloram-phenicol,	<5 (11) each
Clindamycin, Rifampin, Acyclovir, AmphotericinB,	

* Values indicate numbers (Percentages) of patients

In our study, the MDR organisms were most commonly isolated from the lower respiratory tract (49%) and surgical wounds (22%). *Pseudomonas* was isolated in 22 patients and *Acinetobacter* in 21.

### Clinical outcomes:

Overall mortality was 52% and 40% of patients died or had to be discharged before an eight-day course. Among patients who received at least eight days of intravenous polymyxin B, 67% patients with initial septic shock and 62% with severe sepsis survived and recovered.

Acute renal failure developed in only two of the 45 patients treated with polymyxin B (4%). Two patients, a 69-year-old man with esophageal cancer and a 36-year-old lady with vasculitis-induced intracranial bleed developed acute renal failure following treatment with polymyxin B. Acute renal failure developed on day 5 of polymyxin B therapy in the former patient and on day 3 in the later. They died respectively two and nine days after developing acute renal impairment. The mean dose of polymyxin B used was 0.85 × 10^6^ units. The mean rise in creatinine level was 0.6 compared to negative 0.1 in patients without renal failure. None of the four patients with background chronic renal impairment developed any acute deterioration after receiving polymyxin B at mean dose of 1.38 × 10^6^ units.

### Microbiologic outcome:

Repeat microbiologic culture was obtained in 55% of patients. Thirty-two percent of them cleared the target MDR organism, 56% revealed either a different species or the same organism with sensitivity toward safer broad-spectrum antibiotics. Among patients who failed to clear the target organism, 72% had anatomically uncontrollable surgical site infection and 20% had nosocomial pneumonia. Survival among the patients who received at least eight days of therapy was 67% in the septic shock group and 62% in the nonseptic shock group.

## Discussion

Our study is unique as there is very limited data on the efficacy and safety of polymyxin B in critically ill patients with severe sepsis and septic shock. Polymyxin E, that is, colistin has a large body of evidence,[12–18] studies involving polymyxin B[19–20] are limited and our study has the largest number of patients. All patients in our study were critically ill and a majority had severe sepsis and septic shock at time of starting therapy with polymyxin B pointing toward a good efficacy in this group of patients.

The relative slow development of safe and new antibiotics[21] has prompted renewed interest in older agents like the polymyxins which were discontinued from clinical practice due to early reports of nephrotoxicity. However, the absence of a unanimous definition of acute renal failure until recently and the significant variation in the incidence of nephrotoxicity shown by Falagas *et al*, in their review of literature on polymyxins from 1960–2005[13] call for critical enquiry into the issue. Reported incidence of nephrotoxicity in the earlier literature[22–25] varied widely between 20–25% and up to 100% of recipients. Recent Western estimates on nephrotoxicity have been much more moderate with 10–14%[19,20] for polymyxin B and 24% for colistimethate sodium.[13] Sepsis itself has adverse effects on renal function[28] and hence becomes a confounding factor in assessing the nephrotoxicity of polymyxins when therapy is initiated as a last resort in advanced stage of septicemia.

Nosocomial sepsis with septic shock has a very high mortality and an acceptable clinical and microbiologic improvement was obtained with polymyxin B in our study. Overall mortality was 52% in our study. Forty-two percent of patients were in septic shock before initiation of polymyxin B. Polymyxin B was started after results of cultures were obtained in most of the patients with sepsis. Late use of polymyxin B might have blunted its efficacy as definite prognostic advantage is derived with early use of appropriate antibiotics in sepsis.[27] Previous studies with polymyxin B have shown mortality between 20–48%. Mortality in respiratory tract infections treated with polymyxin B[19] is about 48%, and in the study published by Ouderkirk *et al*,[20] the overall mortality was 20%. However, data as to number of patients in shock is not available, so comparative conclusions on efficacy is difficult. Microbiologic response with polymyxin B in our study is also comparable to the efficacy reported in the study by Ouderkirk.

Our limitation is the lack of a comparative cohort treated with other antibiotics. The retrospective nature of this study is also a drawback. Hence, as the stigmata of nephrotoxicity associated with the use of polymyxin B is being questioned worldwide, we need to consider it early in severe nosocomial sepsis where MDR organisms are suspected, with careful monitoring of renal function.

## References

[1] Fridkin SK, Gaynes RP (1999). Antimicrobial resistance in intensive care units. Clin Chest Med.

[2] Fridkin S (2001). Increasing prevalence of antimicrobial resistance in intensive care units. Crit Care Med.

[3] Quinn JP (1998). Clinical problems posed by multiresistant nonfermenting gram-negative pathogens. Clin Infect Dis.

[4] McGowan JE (2001). Economic impact of antimicrobial resistance. Emerg Infect Dis.

[5] Silveira F, Fujitani S, Paterson DL (2004). Antibiotic-resistant infections in the critically ill adult. Clin Lab Med.

[6] Evans ME, Feola DJ, Rapp RP (1999). Polymyxin B Sulfate and Colistin: Old antibiotics for emerging multiresistant gram-negative bacteria. Ann Pharmacother.

[7] Hermsen ED, Sullivan CJ, Rotschafer JC (2003). Polymyxins: Pharmacology, pharmacokinetics, pharmacodynamics and clinical applications. Infect Dis Clin N Am.

[8] Price DJ, Graham DI (1970). Effects of large doses of colistin sulfomethate on renal function. Br Med J.

[9] Wolinsky E, Hines JD (1963). Neurotoxic effects of colistin in patients with renal disease. N Engl J Med.

[10] Dellinger RP, Carlet JM, Masur H, Gerlach H, Calandra T, Cohen J (2004). Surviving sepsis campaign guidelines for management of severe sepsis and septic shock. Crit Care Med.

[11] Levin AS, Barone AA, Pence J, Santos MV, Marianho IS, Arruda EA (1999). Intravenous colistin as therapy for nosocmial infections caused by multidrug-resistant *Pseudomonas aeruginosa* and *Acinetobacter baumanii*. Clin Infect Dis.

[12] Beringer P (2001). The clinical use of colistin in patients with cystic fibrosis. Curr Opin Pulm Med.

[13] Falagas ME, Kasiakou SK, Tsiodras S, Michalopoulos A (2006). The use of intravenous and aerosolized polymyxins for the treatment of infections in critically ill patients: A review of recent literature. Clin Med Res.

[14] Falagas ME, Kasiakou SK (2005). Colistin: The revival of polymyxins for the management of multiresistant gram-negative bacterial infections. Clin Infect Dis.

[15] Garnacho-Montero J, Ortiz-Leyba C, Jimenez-Jimenez FJ, Barrero-Almodovar AE, Garcia-Garmendia JL, Bernabeu-Wittel IM (2003). Treatment of multidrug-resistant *Acinetobacter baumanii* ventilator associated pneumonia (VAP) with intravenous colistin: A comparison with imipenem susceptible VAP. Clin Infect Dis.

[16] Hamer DH (2000). Treatment of nososcomial pneumonia and tracheobronchitis caused by multidrug-resistant *Pseudomonas aeruginosa* with aerosolized colistin. Am J Respir Crit Care Med.

[17] Linden PK, Kusne S, Coley K, Fontes P, Kramer DJ, Paterson D (2003). Use of parenteral colistin for the treatment of serious infection due to antimicrobial-resistant *Pseudomonas aeruginosa*. Clin Infect Dis.

[18] Michalopoulos AS, Tsiodras S, Rellos K, Mentzelopoulos S, Falagas ME (2005). Colistin treatment in patients with ICU-acquired infections caused by multiresistant Gram-negative bacteria: The renaissance of an old antibiotic. Clin Microbiol Infect.

[19] Ouderkirk JP, Nord JA, Turett GS, Kislak JW (2003). Polymyxin B nephrotoxicity and efficacy against nosocomial infections caused by multiresistant gram-negative bacteria. Antimicrob Agents Chemother.

[20] Sobieszczyk ME, Furuya EY, Hay CM, Pancholi P, Della-Latta P, Hammer SM (2004). Combination therapy with polymyxin B for the treatment of multidrug-resistant Gram-negative respiratory tract infections. J Antimicrob Chemother.

[21] Spellberg B, Powers JH, Brass EP, Miller LG, Edwards JE (2004). Trends in antimicrobial drug development implications for the future. Clin Infect Dis.

[22] Koch-Weser J, Sidel VW, Federman EB, Kanarek P, Finer DC, Eaten AE (1970). Adverse effects of sodium colistimethate-manifestations and specific reaction rates during 317 courses of therapy. Ann Intern Med.

[23] Falagas ME, Kasiakou SK (2006). Toxicity of polymyxins: A systematic review of evidence from old and recent studies. Crit Care.

[24] Horton J, Pankey MD (1982). Polymyxin B, colistin and sodium colistimethate. Med Clin N Am.

[25] Nord NM, Hoeprich PD (1964). Polymyxin B and colistin. N Engl J Med.

[26] Rivers E, Nguyen B, Havstad S, Ressler J, Muzzin A, Knoblich B (2001). Early goal-directed therapy in the treatment of severe sepsis and septic shock. N Engl J Med.

[27] Nguyen HB (2007). Implementation of a bundle of quality indicators for the early management of severe sepsis and septic shock is associated with decreased mortality. Crit Care Med.

[28] Schrier RW, Wang W (2004). Acute renal failure and sepsis. N Engl J Med.

